# Adiposity Related Brain Plasticity Induced by Bariatric Surgery

**DOI:** 10.3389/fnhum.2019.00290

**Published:** 2019-08-27

**Authors:** Michael Rullmann, Sven Preusser, Sindy Poppitz, Stefanie Heba, Konstantinos Gousias, Jana Hoyer, Tatjana Schütz, Arne Dietrich, Karsten Müller, Mohammed K. Hankir, Burkhard Pleger

**Affiliations:** ^1^IFB Adiposity Diseases, Leipzig University Medical Centre, Leipzig, Germany; ^2^Department of Nuclear Medicine, University Hospital Leipzig, Leipzig, Germany; ^3^Department of Neurology, Max Planck Institute for Human Cognitive and Brain Sciences, Leipzig, Germany; ^4^Collaborative Research Centre 1052 Obesity Mechanisms, University of Leipzig, Leipzig, Germany; ^5^Department of Neurology, BG University Hospital Bergmannsheil, Ruhr-University Bochum, Bochum, Germany; ^6^Department of Neurosurgery, BG University Hospital Bergmannsheil, Ruhr-University Bochum, Bochum, Germany; ^7^Department of Behavioral Epidemiology, Technische Universität Dresden, Dresden, Germany; ^8^Department of Psychology, Technische Universität Dresden, Dresden, Germany; ^9^Department of Bariatric Surgery, University Hospital Leipzig, Leipzig, Germany; ^10^Department of Experimental Surgery, University Hospital Würzburg, Würzburg, Germany

**Keywords:** adiposity, magnetic resonance imaging, brain plasticity, bariatric surgery, gastric-bypass surgery

## Abstract

Previous magnetic resonance imaging (MRI) studies revealed structural-functional brain reorganization 12 months after gastric-bypass surgery, encompassing cortical and subcortical regions of all brain lobes as well as the cerebellum. Changes in the mean of cluster-wise gray/white matter density (GMD/WMD) were correlated with the individual loss of body mass index (BMI), rendering the BMI a potential marker of widespread surgery-induced brain plasticity. Here, we investigated voxel-by-voxel associations between surgery-induced changes in adiposity, metabolism and inflammation and markers of functional and structural neural plasticity. We re-visited the data of patients who underwent functional and structural MRI, 6 months (*n* = 27) and 12 months after surgery (*n* = 22), and computed voxel-wise regression analyses. Only the surgery-induced weight loss was significantly associated with brain plasticity, and this only for GMD changes. After 6 months, weight loss overlapped with altered GMD in the hypothalamus, the brain’s homeostatic control site, the lateral orbitofrontal cortex, assumed to host reward and gustatory processes, as well as abdominal representations in somatosensory cortex. After 12 months, weight loss scaled with GMD changes in right cerebellar lobule VII, involved in language-related/cognitive processes, and, by trend, with the striatum, assumed to underpin (food) reward. These findings suggest time-dependent and weight-loss related gray matter plasticity in brain regions involved in the control of eating, sensory processing and cognitive functioning.

## Introduction

The incidence of obesity is dramatically increasing worldwide reaching a pandemic level and rendering obesity one of the most challenging health burden of current and future times.

Obese individuals often present comorbidities reaching from cardiovascular and endocrinological diseases to cancer (Dixon, 2010; Ebbert et al., 2014). Impaired cognitive performance and accelerated brain atrophy (Debette et al., 2010; Ho et al., 2010), together with a higher risk of dementia (Knopman et al., 2011; Tosto and Reitz, 2013) indicate negative influences on mental health.

Craving and overconsumption are crucial behavioral features of individuals with obesity. Craving may result from enhanced predictive activity in reward-associated brain regions, whereas during food consumption, reward responses seem to be attenuated probably supporting compensatory overconsumption (Wang et al., 2001; Stice et al., 2008; Johnson and Kenny, 2010). Dysfunctional dopamine release in regions such as the nucleus accumbens, the dorsal striatum and the medial prefrontal cortex seems to have a mediating role in obesogenic effects (Geiger et al., 2008, 2009; Zhang et al., 2015). But dopamine mediates not only food reward suggesting that an aberrant food-related behavior in obese individuals transfers to other reward-related behaviors, such as those associated with monetary incentives (Amlung et al., 2016).

During eating, reward regions permanently interact with the hypothalamus to adjust hedonic responses to current homeostatic demands. In the obese brain, these interactions seem to weaken, and hedonism overpowers homeostasis (Davis et al., 2007; Bryant et al., 2008). Cross-sectional brain imaging studies provide supportive evidence for body weight related functional and structural alteration of hedonic (Wang et al., 2001; Stoeckel et al., 2008; Stice et al., 2010; Mueller et al., 2014) and homeostatic brain regions (Horstmann et al., 2011). But an increased weight also overlapped with executive control regions (e.g., dorsolateral prefrontal cortex, DLPFC) and regions involved in emotional processing (e.g., the amygdala; Jagust et al., 2005; Walther et al., 2010; Debette et al., 2011; Horstmann et al., 2011) suggesting influences on various brain systems guiding everyday eating behavior. An understanding of how this behavior and corresponding brain processes change over time while individuals gain or lose weight may help to develop new therapeutic strategies against obesity.

Bariatric surgery has been established as the gold standard for the treatment of morbid obesity [i.e., body mass index (BMI), BMI ≥ 40 kg/m^2^]. Over the 1st year after surgery, patients experience the largest weight loss with gastric bypass surgery showing the strongest effect on the BMI (i.e., loss of 32%; Sjöström et al., 2004; Robinson, 2009; Gianos et al., 2012). At the same time, energy balance and hence cardiovascular risk factors, such as diabetes and hypertension are progressively improving (Sjöström, 2013). These positive effects on body weight and health suggest the potential reversibility of central homeostatic and hedonic dysfunctions as well as corresponding adiposity-related structural and functional brain alterations.

Only few brain imaging studies to date investigated the influence of bariatric surgery on brain function and structure. Using magnetic resonance imaging (MRI) together with voxel-based morphometry (VBM), surgery-induced brain plasticity was observed not only in hedonic (e.g., striatum), homeostatic (e.g., hypothalamus) and executive control regions (e.g., DLPFC), but across the entire gray (Rullmann et al., 2018) and white matter compartments (Tuulari et al., 2016). This suggests much more far-reaching behavior adaptations than only those involved in the control of food consumption.

Rullmann et al. (2018) showed that the higher the BMI loss after surgery, the larger MRI-based changes of gray and white matter densities (GMD/WMD) across the entire brain were. No other markers of changes in adiposity, metabolism or inflammation significantly scaled with surgery-induced brain plasticity (Rullmann et al., 2018). These findings render the degree of adiposity (Gurka et al., 2018) a potential marker for surgery-induced widespread white and gray matter plasticity. Significance of this correlation, however, may have been constituted only by some voxels, whereas other voxels may have been less meaningful or even irrelevant. Clusters of significantly correlated voxels may, furthermore, overlap with distinct brain regions providing a more fine-grained picture of adiposity-related influences on the brain induced by bariatric surgery.

To unveil voxel-wise, and potentially region-specific associations with surgery-induced changes in adiposity, metabolism and inflammation, we re-analyzed MRI data from our recent longitudinal study (Rullmann et al., 2018). We computed voxel-by-voxel regression analyses including individual surgery-induced changes in gray matter (i.e., GMD), white matter (i.e., WMD and diffusion tensor imaging, or DTI, to assess fractional anisotropy, FA) and spontaneous neural activity (regional homogeneity, ReHo, and amplitudes of low frequency fluctuation, ALFF). Corresponding surgery-induced improvements in markers of adiposity, metabolism and inflammation [i.e., body weight (kg), fat mass (%), serum high-density lipoproteins (HDL), low-density lipoproteins (LDL), triglyceride, HbA1c, CrP, thyroid-stimulating hormone (TSH)] were included as interacting covariates.

Contrarily to the widespread plasticity, we found in our previous data analyses (Rullmann et al., 2018), we here hypothesized a more detailed picture of surgery-induced clinical improvements and associated structural (i.e., GMD, WMD, FA) and functional plasticity (ALFF, ReHo). We assumed to identify a network consisting of the dorsal striatum, involved in reward processing (Stice et al., 2008), the insular cortex, assumed to host gustatory processes (Tuulari et al., 2016; Rullmann et al., 2018), the hypothalamus, as the main homeostatic control site (van de Sande-Lee et al., 2011; Hollmann et al., 2013; Schlogl et al., 2016), as well as executive control regions in the DLPFC (Hollmann et al., 2013).

## Materials and Methods

### Patients

The data for the present analyses was obtained from the same 27 morbidly obese patients (21 females, age 51.1 ± 9.6 years, BMI 47.8 ± 5.5 kg/m^2^) who participated in our previous study (Rullmann et al., 2018). Contraindications to MRI were exclusion criteria (see Supplementary Information in Rullmann et al., 2018 for a detailed list). Patients gave written informed consent. Table 1 lists clinical parameters such as (BMI, kg/m^2^), body weight (kg), fat mass (%), cholesterol serum levels (mmol/l), HDL (mmol/l), LDL (mmol/l), triglycerides (mmol/l), as well as glycated hemoglobin (HbA1c, %) which indexes the 3-months average plasma glucose concentration for the three time points (prior to surgery, after 6 and 12 months). C-reactive protein (CrP, mg/l) was used as a measure of peripheral inflammation. As a more global marker of metabolism, we measured blood levels of (TSH, mU/l). Patients were investigated prior to, and 6 months after laparoscopic proximal Roux-en-Y gastric bypass. This procedure involves building a small gastric pouch which is anastomosed to a Roux limb of the jejunum. Twenty-two out of the 27 patients (18 females, age 52.6 ± 8.5 years, BMI 47.6 ± 5.5 kg/m^2^) participated in the same experiments 1 year after surgery. The study was carried out in accordance with the recommendation of the ethics guideline, ethics committee of the medical faculty, University of Leipzig. The protocol was approved by the same ethics committee. All patients gave written informed consent in accordance with the Declaration of Helsinki. MRI data was acquired with a 3-Tesla Siemens VERIO system (Siemens, Erlangen, Germany). No hardware or software upgrades were applied during the study. Patients were placed in the scanner in supine position. The MRI data is stored on a computer server located at the Max-Planck-Institute for Human Cognitive and Brain Sciences and the clinical data on a server located at the University Hospital Leipzig, Department of Bariatric Surgery. The data is available on request directed to MR.

**Table 1:** The table presents body mass index (BMI), gender, weight, blood parameters [cholesterol, high-density lipoproteins (HDL), low-density lipoproteins (LDL), triglyceride, glycated hemoglobin (HbA1c), C-reactive protein (CrP), thyroid-stimulating hormone (TSH)], and fat mass (%).

Subject	Age	Gender	Weight			BMI			Cholesterol			HDL			LDL			Triglyceride			HbA1c			CrP			TSH			Fat mass		
			pre	½ year	1 year	pre	½ year	1 year	pre	½ year	1 year	pre	½ year	1 year	pre	½ year	1 year	pre	½ year	1 year	pre	½ year	1 year	pre	½ year	1 year	pre	½ year	1 year	pre	½ year	1 year
#1	60	♀	147	105	100	58.7	42.1	40.2	4.84	4.8	4.69	1.61	1.55	1.73	2.58	2.68	2.84	1.33	0.86	1.2	6.46	5.87	5.31	19.5	3.91	2.46	1.04	0.406	0.824	62.2	53.4	48.7
#2	54	♀	123	105	101	44.6	38.1	36.6	4.62	4.29	3.89	1.65	1.55	1.76	2.61	2.38	1.99	1.52	1.19	1.18	4.97	5.1	5.25	8.3	6.18	2.86	2.28	3.18	1.75	N/A	N/A	48.1
#3	49	♂	165	126	113	44.2	33.7	30.4	3.95	3.28	4.35	1.09	1.16	1.31	2.25	1.91	2.96	1.32	0.92	0.82	5.62	5.51	5.3	7.05	1.23	1.48	0.7	0.731	0.64	44.9	33.3	27.3
#4	54	♀	104	79.9	76	44.5	34.1	32.5	4.64	5.09	4.51	1.17	1.44	1.44	3.23	3.19	2.78	1.65	1.48	1.02	5.06	4.83	4.99	5.8	2.61	1.73	0.712	2.63	0.637	49.4	32.9	27.2
#5	59	♀	135	105	95.8	47.3	37.3	33.9	5.99	4.63	5.1	2.27	2.16	2.6	3.35	2.47	2.47	0.8	0.59	0.87	5.28	5.03	5.08	21.4	2.87	1.87	0.787	2.02	1.9	59.9	44.4	39.7
#6	60	♀	120	97.7	96.6	38.7	31.5	31.2	4.95	5.08	4.3	0.98	1.54	1.73	2.96	2.5	1.88	2.92	3.75	1.9	5.6	5.01	5.19	3.77	1.5	0.89	0.65	0.763	0.919	52.8	41.1	N/A
#7	44	♀	143	99.2	87.5	52.3	36.4	32.1	4.53	4.66	4.17	0.83	1.3	1.72	2.95	2.85	2.23	1.42	1.05	0.63	5.45	5.24	5.42	24.7	1.03	0.99	1.22	1.08	0.764	49.0	36.6	38.7
#8	45	♀	161	129	127	47.6	38.1	37.4	3.48	3.52	3.93	1	1.27	1.47	2.03	2	2.39	0.89	1.28	1.19	5.35	4.69	5.07	13.9	8.44	2.26	0.563	1.56	1.09	62.7	39.2	34.0
#9	60	♀	120	103	102	48.1	41.3	40.7	3.72	4.51	5	1.05	1.49	1.26	2.27	2.54	3.04	1.54	1.8	3.05	6.11	5.52	5.66	6.1	2.11	2.05	1.14	0.696	1.24	65.1	54.0	50.5
#10	38	♀	154	123	103	54.0	43.0	36.2	4.98	4.19	3.83	1.03	1.21	1.43	3.51	2.62	2.22	1.83	0.9	1.06	4.97	4.67	5.16	6.53	1.76	0.68	0.75	0.828	0.766	59.4	50.2	38.4
#11	49	♀	138	112	104	52.7	42.6	39.5	4.46	4.15	4.29	1.3	1.81	1.84	2.66	2.03	2.17	1.17	1.44	1.62	5.21	4.85	4.88	9.78	3	1.14	2.22	1.87	2.04	63.1	49.1	43.1
#12	39	♀	121	88	87.5	42.8	31.2	31.0	5.52	3.04	3.47	1.14	1.21	1.08	3.49	1.7	2.18	2.63	0.58	0.7	10.5	5.25	5.73	1.8	0.3	1.71	0.271	0.543	0.598	51.4	22.6	31.7
#13	66	♂	127	96	82.8	40.1	30.3	26.1	4.04	3.6	3.97	1.29	1.4	2.15	2.32	2.06	1.67	0.97	0.74	0.81	7.49	6.18	6.25	0.6	0.45	0.3	0.677	0.543	1	34.4	25.1	17.2
#14	54	♀	116	92.9	89.1	41.0	32.9	31.6	6.01	5.71	5.91	0.99	1.68	1.68	4.4	3.78	4.04	0.98	1.06	0.95	6	5.25	5.62	11.3	6.96	2.28	1.47	1.7	1.6	53.4	39.0	39.9
#15	60	♀	150	130	120	58.7	50.9	46.7	4.88	N/A	3.67	1.17	N/A	0.82	3.17	N/A	2.25	1.28	N/A	1.16	5.59	N/A	5.5	16.2	N/A	30.2	3.73	N/A	2.33	62.1	60.5	57.4
#16	30	♀	120	89.1	80.2	44.2	32.7	29.5	3.2	3.43	2.81	1.13	1.53	1.43	1.72	1.62	1.08	0.94	1.35	0.55	5.19	5.12	5.13	7.84	2.34	47.5	2.55	2.56	0.847	60.5	40.6	36.2
#17	52	♀	138	112	107	45.1	36.6	35.0	4.46	4.01	3.73	1.52	1.56	1.62	2.55	2.11	1.66	1.12	1.36	1.32	5.17	5.27	5.22	4	0.95	1.43	1.33	1.57	1.09	57.2	47.2	38.5
#18	55	♀	115	91.7	101	42.6	34.1	37.5	4.44	3.88	4.74	1.35	1.37	1.37	2.87	2.34	2.96	1.53	1.74	1.42	5.78	5.42	5.75	5.33	1.06	3.06	2.33	1.14	1.16	43.5	30.7	37.5
#19	55	♂	149	105	N/A	46.0	32.5	N/A	2.99	3.21	N/A	0.92	1.05	N/A	1.91	1.98	N/A	0.91	0.95	N/A	6.35	7.03	N/A	0.84	0.3	N/A	1.34	3.25	N/A	41.1	20.1	N/A
#20	54	♀	173	146	133	47.5	40.0	36.6	4.88	N/A	4.52	1.16	N/A	1.35	3.33	N/A	2.75	1.83	N/A	1.5	5.73	5.71	5.96	2.41	2.78	1.12	1.52	0.533	1.08	N/A	N/A	N/A
#21	55	♂	131	109	104	44.7	37.4	35.4	4.72	4.39	5.08	1.02	0.97	1.06	3.2	2.96	3.26	2.03	1.31	0.98	8.21	7.47	7.07	7.79	4.19	4.77	1.75	1.95	1.8	31.5	21.9	25.9
#22	59	♀	112	89.7	88	47.3	37.8	37.1	4.31	3.14	3.25	1.17	1.43	1.49	2.52	1.36	1.45	2.31	0.84	1.09	9.11	7.01	7.07	4.76	0.91	1.27	4.24	2.36	2.63	N/A	N/A	N/A
#23	52	♂	161	127	118	50.8	40.1	37.1	3.82	4.24	3.75	1.03	1.58	1.57	2.42	2.39	2.09	0.76	0.65	0.62	5.34	5.33	5.18	8.93	5.65	3.49	0.956	1.42	1.51	49.2	31.3	27.7
#24	27	♀	166	132	N/A	56.2	44.7	N/A	2.65	2.92	N/A	0.86	1.05	N/A	1.52	1.59	N/A	0.96	0.87	N/A	5.55	5.29	N/A	22.6	14.1	N/A	2.28	1.86	N/A	59.8	47.4	N/A
#25	42	♀	129	87.1	N/A	44.5	30.1	N/A	5.21	4.6	N/A	1.26	1.27	N/A	3.54	2.77	N/A	2.91	0.82	N/A	5.76	5.23	N/A	15.1	2.16	N/A	2.23	2.88	N/A	54.0	30.4	N/A
#26	39	♂	175	130	N/A	55.1	41.2	N/A	5.77	4.46	N/A	1.04	0.85	N/A	4.1	3.12	N/A	1.17	0.96	N/A	5.62	5.22	N/A	5.92	3.65	N/A	2.26	2.75	N/A	52.6	33.2	N/A
#27	57	♀	139	109	N/A	51.7	40.5	N/A	4.95	3.87	N/A	1.27	1.27	N/A	3.2	2.27	N/A	1.31	1.19	N/A	5.91	5.44	N/A	12.5	7.4	N/A	0.579	0.566	N/A	59.7	42.5	N/A

*Red indicates outliers (i.e., any changes over time beyond mean plus/minus two times the standard deviation)*.

### MRI Parameters for Voxel-Based Gray Matter Morphometry (GMD/WMD)

We applied a T1-weighted three-dimensional magnetization-prepared rapid gradient echo (MP-RAGE) sequence (Mugler and Brookeman, 1990) in sagittal slice orientation [inversion time (TI) = 900 ms; repetition time (TR) = 1,300 ms; echo time (TE) = 2.98 ms; readout pulse flip angle, *α* = 9°; bandwidth = 238 Hz/pixel; image matrix = 256 × 240; field of view (FOV) = 256 × 240 mm^2^; nominal spatial resolution = 1 × 1 × 1 mm^3^; 1 average].

### MRI Parameters for Resting-State fMRI (ReHo, ALFF)

Patients were instructed to fixate a cross in the middle of a screen throughout the “resting-state” session. We darkened the MRI cabinet. We assessed 238 echo planar (EPI) images [voxel size of 3 × 3 × 4.8 mm^3^, TR of 2,000 ms, TE of 30 ms, slice thickness (ST) of 4 mm]. One patient canceled data acquisition. That is why data of only 26 patients were available for respective ReHo/ALFF analyses.

### Parameters for Diffusion-Weighted MRI (FA)

At the end of the session, we acquired whole-brain diffusion-weighted images (twice-refocused spin echo echo-planar-imaging sequence, 64 axial slices, 2-mm thickness, no gap, TE = 88 ms, TR = 12.4 s, *α* = 90°, bandwidth = 1,684 Hz/pixel, FOV = 220 × 220 mm^2^, 110 × 110 image matrix, GRAPPA, acceleration factor 2; Reese et al., 2003). We applied a *b*-value of 1,000 s/mm^2^ and 60 diffusion-encoding gradient directions. As anatomical reference for offline motion correction, we acquired seven images without diffusion weighting (*b*-value 0 s/mm^2^), one at the beginning and one after each block of 10 images.

### Data Analyses

### Surgery-Induced Changes in Adiposity (Body Weight, Fat Mass)

Since body length did not change over 1 year, we here used body weight (kg) and fat mass (%) instead of the BMI as measures of adiposity. Both measures (weight and fat mass) for each of the three time points were applied to the within-subject two-tailed repeated-measures analysis of variance (ANOVA; significance threshold *p* = 0.05) followed by Bonferroni-corrected *post hoc* paired *t*-tests (p adjusted to *p* < 0.025). We also tested for a significant correlation between changes in body weight and fat mass using Pearson’s correlation analysis together with Bonferroni-correction (*p* < 0.025 indicated significance).

In the next step, changes in clinical parameters (i.e., deltas), as listed in Table 1, including body weight (kg) and fat mass (%) were considered as covariates for one-sample *t*-tests with functional (ALFF, ReHo) and structural MRI markers (GMD, WMD, FA) of surgery-induced brain plasticity. To rule out, that significant correlations resulted from few outliers, we excluded outliers (i.e., any value beyond mean plus/minus two times the standard deviation, see red-colored data in Table 1).

### Preprocessing of T1-Weighted MRI Data for VBM

Prior to each processing step, the authors MR and BP visually checked T1-weighted MR images for motion artifacts using the CheckReg option under SPM8 (Wellcome Trust Centre for Neuroimaging, UCL, London, UK) running under Matlab 7 (Mathworks, Sherborn, MA, USA). Anterior/posterior commissure, frontal pole, occipital pole and the cranial peak of the sulcus centralis were used as anatomical landmarks across images. We found no differences in image quality across the three time points and no patient was excluded due to artifacts. We transformed images using the VBM8 toolbox and applied them to the longitudinal approach including segmentation and bias-correction. We used the Diffeomorphic Anatomical RegisTration using Exponentiated Lie algebra (DARTEL) technique (Ashburner, 2007) with a pre-defined tissue probability map registered to the Montreal Neurological Institute (MNI) template to normalize images. Data was then smoothed using a Gaussian filter of 8 mm full width at half maximum (FWHM). In agreement with general practice, we used the term GMD and WMD to describe the normalized gray and white matter probability values, respectively.

### Preprocessing of Resting-State fMRI for ReHo and ALFF Analyses

We removed the first four volumes to guarantee steady state of blood oxygenation-level-dependent (BOLD) signals. All other 234 images were applied to SPM8. After each processing step, images were visually checked using SPM’s CheckReg function with the same landmarks as for T1-weighted images. Images were slice time corrected and realigned. Averaged scan-to-scan deviations during realignment in all patients indicated appropriately small displacements (x, y, z in mm: pre 0.0003, 0.0002, 0.0001, after 6 months 0.0001, 0.00003, 0.0003, after 12 months 0.006, 0.005, 0.004; pitch, roll, yaw in rad: pre 0.0000005, 0.0000006, 0.000004 after 6 months 0.0000002, 0.00002, 0.000001, after 12 months 0.000002, 0.000007, 0.000006). Finally, images were spatially normalized to the MNI template. We found no differences in image quality across the three time points and no patient was excluded due to artifacts.

ReHo was computed using the REST toolbox for SPM8^1^ and for ALFF data was applied to the FMRIB Software Library (FSL^2^).

To assess ReHo, we computed Kendall’s coefficient of concordance as a measure of the similarity of a voxel’s ranked time series to its nearest 27 neighbor voxels (Zang et al., 2004).

For assessing ALFF, images were spatially smoothed using a Gaussian filter of 8 mm FWHM. ALFF was computed for the low frequency range, from 0.01 Hz to 0.08 Hz. We transformed the voxel-wise time series to the frequency domain and computed the power spectrum. The square of the amplitude of this frequency component is proportional to the power of a given frequency. That is why we computed the square root at each frequency of the power spectrum and obtained the averaged square root across 0.01–0.08 Hz at each voxel (Zou et al., 2008).

### Preprocessing of Diffusion Tensor MRI for FA Analyses

We checked the quality of diffusion-weighted images using DTIPrep (Oguz et al., 2014) using the same anatomical landmarks as for the other MRI modalities. We found no differences in image quality across the three time points and no patient was excluded due to artifacts. For further processing we applied images to the FMRIB Software Library (FSL; Smith et al., 2004) and LIPSIA^3^ (Lohmann et al., 2001). Images were motion corrected using rigid-body transformation (Jenkinson et al., 2002), based on the anatomical reference images. This was combined with global registration to the T1-weighted images. After skull-stripping images were co-registered to the standard MNI template. We applied rotation parameters to correct gradient direction of each image. We interpolated registered images of individual anisotropy, using an isotropic voxel resolution of 1 mm. The diffusion tensor was fitted to each voxel. FA was calculated from the eigenvalues of the diffusion tensor. Voxel-wise FA using Tract-Based Spatial Statistics (TBSS; Smith et al., 2006) was conducted with FSL (Smith et al., 2004). We applied nonlinearly coregistered to map FA images onto the FMRIB58_FA standard-space skeleton and finally thresholded images at 0.2.

### Group-Level Analyses of GMD, WMD, ReHo, ALFF, FA

Voxel-wise one-sample *t*-tests were used to analyze changes of GMD, WMD, ReHo, ALFF, FA in association with clinical improvements (see Table 1) including reductions of body weight (kg) and fat mass (%). We subtracted normalized brain images of the pre-session from their corresponding post-session, resulting in brain maps of absolute changes (i.e., 6 months after surgery—pre, 12 months after surgery—pre). Corresponding changes (i.e., deltas) in clinical parameters were inserted as interacting covariates (one model for each parameter). We also included gender, age and global shifts of GM/WM volume over time as nuisance regressors. Significant levels were Bonferroni-corrected to account for repeated measures (i.e., 6 months after surgery—pre, 12 months after surgery—pre). Accordingly, GMD/WMD clusters were assessed by applying a peak-level threshold of *p* < 0.0005 and a cluster-level threshold of *p* < 0.025, family-wise error (FEW)-corrected. Significant changes of FA, ALFF and ReHo were detected with a voxel threshold of *p* < 0.025, FWE-corrected. To assign group effects to the underlying brain anatomy, we used the Anatomy toolbox for SPM (Eickhoff et al., 2005), as well as the WFU_PickAtlas by Joseph Maldjian from the Functional MRI Laboratory at the Wake Forest School of Medicine (see http://www.nitrc.org/projects/wfu_pickatlas).

## Results

### Changes in Body Weight (kg) and Fat Mass (%) After Surgery

**Figure 1:**
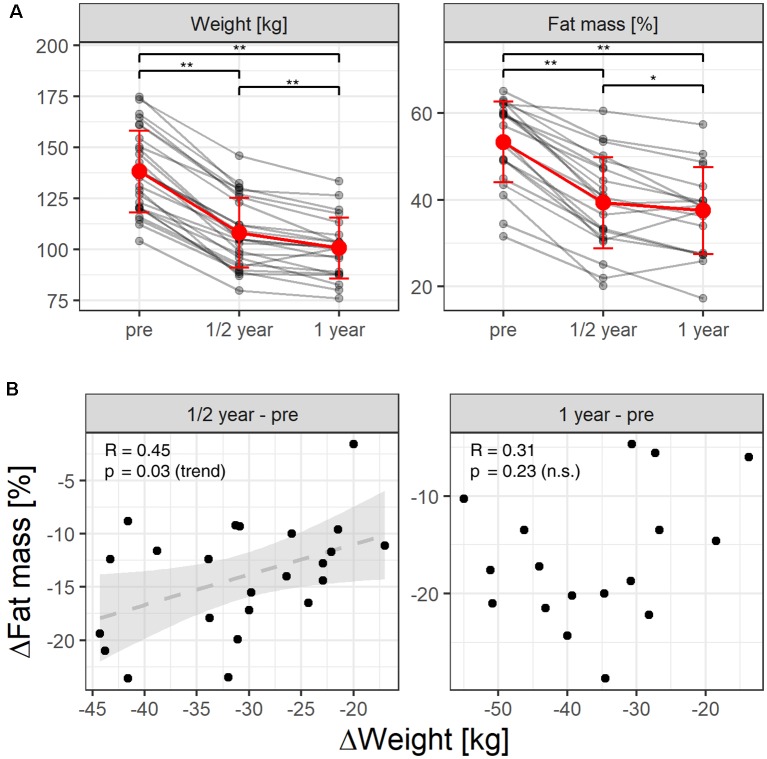
**(A)** Individual weight and fat loss before and after gastric-bypass surgery (after 6 month and after 1 year). Red color indicates the mean over time. The whiskers the standard deviation. *Indicate a *p*-value of *post hoc* *t*-tests < 0.025, **indicate *p* < 0.001. **(B)** Pearson-correlation unexpectedly revealed that reductions in fat mass (%) and reductions in body weight (kg) correlated only by trend over the 1st 6 months after surgery, whereas after 12 months we found no correlation between both adiposity measures.

After surgery, patients presented the expected weight/fat loss (Figure 1A; repeated measures ANOVA, weight: *p* < 2 × 10^−16^, fat mass: *p* = 2.54 × 10^−16^), 6 months (weight: 108.2 ± 17.1 kg; fat mass: 39.3 ± 10.6%) as compared to the weight/fat mass assessed prior to surgery (*n* = 27, 138.2 ± 20 kg, *p* = 6.4 × 10^−12^, 53.4 ± 9.3%, *p* = 9.3 × 10^−8^), as well as 1 year after surgery as compared to prior to surgery (*n* = 22, 100.7 ± 14.9 kg, *p* = 0.0002, 37.5 ± 10%, *p* = 0.017). Loss of body weight (kg) and fat mass (%) were not highly correlated as expected (Figure 1B). Six months after surgery, changes in both adiposity measures correlated only by trend (*p* = 0.03, *r* = 0.45), whereas there was no significant correlation after 12 months (*p* = 0.23, *r* = 0.31). Prior to surgery, six patients suffered from diabetes type 2. After surgery, three patients out of these six recovered from diabetes as indicated by normalized glucose and HbA1c blood levels (see Table 1).

### Weight Loss Associated Alterations of GMD

We only found significant associations between changes in GMD and the surgery-induced weight loss. Other markers of functional (ALFF, ReHo) and structural brain plasticity (FA) remained non-significant. We also found non-significant associations between plasticity markers and fat mass. Also, other possible associations between measures of neural plasticity and any other clinical parameter listed in Table 1 (i.e., deltas of blood parameters of cholesterol, HDL, LDL, triglyceride, HbA1c, CrP, TSH) remained non-significant.

**Figure 2:**
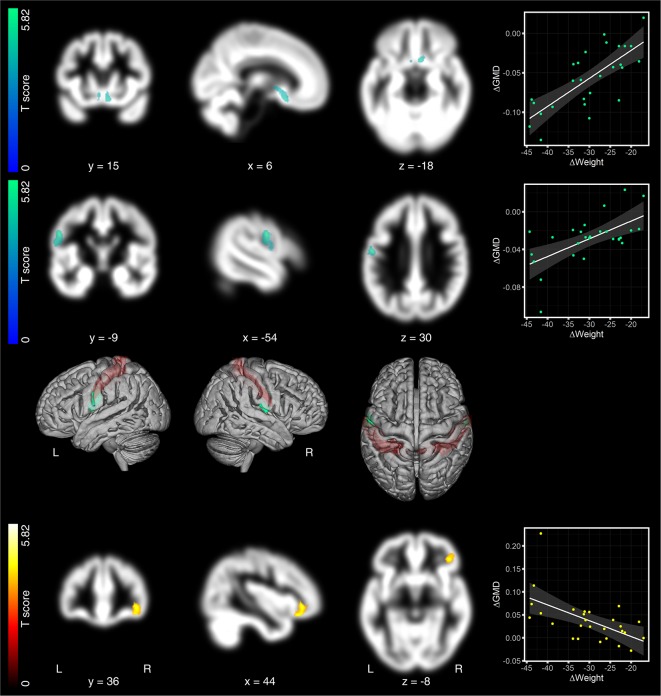
Regression analyses between changes in gray matter density (GMD) maps and weight, 6 months after surgery. The loss of body weight (in kg) from pre to 6 months after surgery was associated with decreased GMD in the hypothalamus (top row) and the left postcentral gyrus (second row). In right postcentral gyrus, directly opposite to the left-sided cluster, we identified another cluster, which did not survive significance threshold. Due to the symmetry of both clusters across both hemispheres, we considered this non-significant cluster, nevertheless, as relevant for the discussion (third row). The only region that presented significantly increased GMD was the right lateral OFC (bottom row). Significant GMD clusters are projected on patients’ mean GMD template. Hot colors indicate positive and cold colors negative associations. GMD effects are presented on a coronal, sagittal, and axial brain slice (from left to right). Coordinates below each brain slice indicate clusters’ peak coordinates in Montreal Neurological Institute (MNI) space. “L” indicates the left, “R” the right brain hemisphere. The color bar on the left side indicates the *T*-scores for the cluster presented in that row. In the 3rd row, we superimposed effects on both postcentral gyri on a rendered brain. Postcentral gyri are colored in red. Scatter plots on the right to the brain slices show the relationship between changes in GMD and changes in weight. Changes in GMD, as shown in the scatter plots, were extracted as the 1st Eigenvariate at *p* = 0.025 family-wise error (FWE), cluster-threshold and *p* = 0.0005, uncorrected peak threshold.

Analysis of associations between changes in GMD and weight loss across the first two time points (6 months after surgery—pre) revealed Bonferroni and FWE-corrected decreased GMD in the hypothalamus (1st peak-level: 6, 15, −18 in mm, MNI coordinates, *T* = 4.95, *p* < 0.0005; cluster-level: *p* = 0.016 FWE-corrected, *k*_E_ = 461 voxels; 2nd peak-level: 6, 8, −6, *T* = 4.6; 3rd peak-level: −3, 6, −6, *T* = 4.41; Figure 2, first row). We identified a second significant cluster of GMD decrease in the left parietal cortex around the central sulcus also consisting of three peaks [1st peak-level: −54, −9, 30, *T* = 4.48, *p* < 0.0005; cluster-level: *p* = 0.006 FWE-corrected, *k*_E_ = 559 voxels, assigned to Brodmann area (BA) 4p/3b, 2nd peak-level: −50, −7, 19, *T* = 4.07, assigned to BA 3a; 3rd peak-level: −57, −1, 12, *T* = 4.41, assigned to BA 3b/44; Figure 2, second row]. In right parietal cortex, directly opposite to the former cluster, we identified another cluster with a single peak which spreads from the parietal operculum into postcentral gyrus. This cluster, however, did not reach cluster significance level (peak-level: 60, −7, 9, *T* = 4.85, *p* < 0.001; cluster-level: *p* = 0.763 FWE-corrected, *k*_E_ = 99 voxels, Figure 2, third row). The only region that presented significant increases in GMD was the right lateral OFC (peak-level: 44, 36, −8, *T* = 5.24, *p* < 0.0005; cluster-level: *p* = 0.020 FWE-corrected, *k*_E_ = 438 voxels; Figure 2, bottom row).

**Figure 3:**
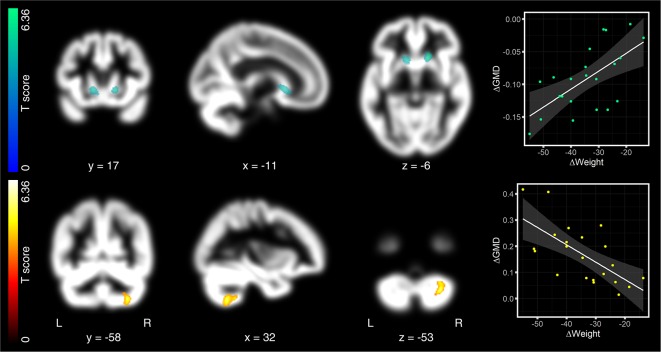
Regression analyses between changes in GMD maps and weight, 12 months after surgery. After 12 months, the loss of body weight was associated with decreased GMD in the left caput caudate. This cluster reached peak significance level, but just missed significant cluster threshold (*p* = 0.029; threshold at *p* = 0.025, FWE-corrected; first row). Directly opposite to this cluster, we also identified the right caput caudate, but below the significant threshold. We, nevertheless, considered both clusters together as relevant for the discussion due to the symmetry of effects. Increased GMD after 12 months was observed in the right cerebellar lobule VII (second row). As in Figure 2, hot colors indicate positive and cold colors negative associations. Significant clusters are projected on patients’ mean GMD template, presented on a coronal, sagittal, and axial brain slice (from left to right). Coordinates below each slice index coordinates of the clusters’ peak in MNI space. “L” indexes the left, “R” the right brain hemisphere. The left-sided color bar shows the *T*-scores for the respective GMD cluster. Right-sided scatter plots present the association between changes in GMD and changes in body weight. As for Figure 2, changes in GMD in the scatter plots were extracted as the 1st Eigenvariate at *p* = 0.025 FWE, cluster-threshold and *p* = 0.0005, uncorrected peak threshold.

After 1 year (pre as compared to 12 months after surgery), we found no GMD decreases surviving corrected FWE-threshold. One cluster in the left caput caudate just missed significance (peak-level: −11, 17, −6, *T* = 5.09, *p* < 0.0005; cluster-level: *p* = 0.029 FWE-corrected, *k*_E_ = 364 voxels; Figure 3, first row). Directly opposite to this cluster, we identified another cluster in right caput caudate. Although peak level was higher as compared to the left cluster, cluster level did not reach significant cluster level (peak-level: 17, 20, −6, *T* = 6.36, *p* < 0.0005; cluster-level: *p* = 0.141 FWE-corrected, *k*_E_ = 236 voxels; Figure 3, first row). We, nevertheless, considered both clusters together as relevant for the discussion due to their symmetry. We also observed significantly increased GMD that overlapped with the right cerebellar lobule VII (peak-level: 32, −58, −53, *T* = 5.53, *p* < 0.0005; cluster-level: *p* = 0.009 FWE-corrected, *k*_E_ = 464 voxels; Figure 3, second row).

## Discussion

Here, we applied three MRI methods to investigate surgery-induced voxel-by-voxel structural and functional plasticity of gray and white matter in association to individual improvements in adiposity, metabolism and inflammation [as indexed by changes in body weight (kg), fat mass (%) and the blood levels of cholesterol, HDL, LDL, triglyceride, HbA1c, CrP, TSH]. Only the loss of body weight (in kg) was significantly associated with surgery-induced brain plasticity, and this only for GMD changes. Due to the longitudinal design of our study, body weight (kg) and the BMI (kg/m^2^) were redundant measures, since body length (m) did not change over time. Unexpectedly, reductions in fat mass (%) and reductions in body weight (kg) correlated only by trend 6 months after surgery (*r* = 0.03), whereas after 12 months we found no correlation between them. Significant brain-associations were only identified for the weight loss, but not for the loss of fat mass suggesting that both markers address different aspects of adiposity.

### Six Months After Surgery

Six months after surgery, reduced body weight overlapped with decreased GMD in the hypothalamus. One of the hypothalamus’s central functional attribution is to receive metabolic feedback from the digestive tract and fat depots to control hedonic responses to food (Hollmann et al., 2013; Schlogl et al., 2016). We found that the higher the individual BMI loss, the higher the loss of GMD in the hypothalamus was. Previous functional MRI studies in patients after bariatric surgery revealed increases in neural activity in the hypothalamus as a response to standardized glucose load (van de Sande-Lee et al., 2011), suggesting a surgery-induced regain in hypothalamic responsivity to sweet and high-calorie food. Although it is generally difficult to infer the functional relevance of GMD changes, the loss in hypothalamic GMD in our study may reflect structural correlates of surgery-induced improved hypothalamic sensitivity to an altered homeostatic demand.

Besides the hypothalamus, we found weight-loss related decreases in GMD in the left parietal cortex, around the central sulcus, comprising the pre- (i.e., BA 4p) and postcentral gyrus (i.e., BA 3a/b). The postcentral gyrus is known to host the primary somatosensory cortex. Its organization is well described by the so-called homunculus (i.e., the little man in the brain), with different sub-regions being involved in processing somatosensory feedback from distinct body parts (i.e., cortical representations; Pleger and Villringer, 2013). The location of GMD effects in the present study overlapped with the representation of the digestive system, suggesting that the loss of GMD relates to adaptation processes of abdominal organs, like the stomach, with associated influences on the somatosensory feedback to corresponding cortical representations. Neighbored BA 4p (p for posterior) in the precentral gyrus is cytoarchitectonically different from the more anteriorily located primary motor cortex, BA 4a. BA 4p seems to be involved in both, motor and somatosensory processing suggesting close functional-anatomical associations to directly neighboring S1 (i.e., BA 3b; Geyer et al., 1996). On the contralateral hemisphere, we also found a cluster in the same topographic location. This cluster did not reach significance, but its symmetric occurrence across both hemispheres, nevertheless, complicates the discussion about a hemispherical dominance of the observed effects.

The last region that presented changes in GMD related to the surgery-induced weight loss was the right lateral orbitofrontal cortex, well known as a key region of the brain’s reward circuity (Noonan et al., 2012). As compared to the hypothalamus and postcentral gyri, lateral OFC presented not decreased but increased GMD. In previous studies, activity in lateral OFC was found to positively correlate with the subjective appeal of high-calorie food in the fasting state, suggesting that fasting biases regions of the brain reward circuitry, such as the lateral OFC, towards high-calorie food (Goldstone et al., 2009). Besides its role in food reward, studies in the late 90th suggest its contribution to taste and smell processing (Small et al., 1997; Zald et al., 1998), suggesting that the lateral OFC, especially in the right hemisphere, plays a role in gustatory processes, too (Small et al., 1997, 1999; Zald et al., 1998; Rolls, 2005). Thus, elevated GMD in lateral OFC may not only reflect altered food reward but also gustatory processing.

### Twelve Months After Surgery

Twelve months after surgery, we also found decreased GMD, not in the hypothalamus as after the first 6 months, but in the caput caudate. The caput caudate belongs to the dorsal striatum. It is a dopamine-rich reward region involved in guiding food consumption and its devaluation when eating beyond satiety (Small et al., 2001). Its activity during the consumption of chocolate milkshake seems to correspond to future gains in body weight in young adolescence females (Stice et al., 2008). Obese individuals, like drug addicts, present attenuated reward responses in the dorsal striatum during the ingestion of food, probably promoting compensatory overconsumption (Wang et al., 2001; Stice et al., 2008; Johnson and Kenny, 2010). The dorsal striatum is permanently exchanging information with the hypothalamus to sustain energy homeostasis. The weight loss related decrease of GMD in the hypothalamus 6 months after surgery, together with the GMD decrease in caput caudate 6 months later, may thus indicate a progressively improving hedonic-homeostatic crosstalk over the 1-year observation period.

Besides weight-loss related decreased GMD, 12 months after surgery, we also found significantly increased GMD that comprised the right cerebellar lobule VII. The higher the BMI-loss, the more GMD increased. The functional attribution of this cerebellar effect is difficult since this region remains less well investigated as the other regions we found. A series of studies investigated the attribution of the cerebellar lobule VII to phonological storage, which is assumed to depend on projections between parietal regions and right caudo-lateral lobule VIIb (Desmond et al., 1997; Chen and Desmond, 2005), specifically during late memory encoding, when encoding and the transfer of verbal items to phonological storage run in parallel (Macher et al., 2014). Whether an increased GMD relates to improved verbal memory capacities or other cognitive gains remains unanswered. These speculations clearly warrant further longitudinal studies combining brain imaging with cognitive tests.

### Limitations

Some important limitations of our study must be taken into consideration. First, our study does not contain control persons, neither lean nor comparably obese, that were investigated over the same observation period with the same clinical or imaging methods. Furthermore, our study does not address how the amelioration of obesity-related comorbidities may relate to differences in neural plasticity. This is due to the small sample size that limits generalization of brain-adiposity associations and analyses of sub-populations, such as those patients that recovered from diabetes after surgery (see the drop of HbA1c levels into normal range in three patients in Table 1). Not only the sample size *per se*, but also the imbalance between male and female patients should be considered as a further limitation. Our sample mainly consisted of female patients. That is why we accounted for gender effects during data analyses to minimize potential confounding influences. Gender-related differences in neural plasticity, however, remain unaddressed by our study. The small sample size may also explain why we found statistical evidence for adiposity-related neural plasticity only for GMD and not for any other plasticity marker or any other clinical parameter.

### Outlook

Future studies should include a larger sample size that allows sub-population analyses. These studies should furthermore combine cognitive tests and metabolic/inflammatory markers with measurements of structural and functional brain plasticity to assess brain representations of integrative cognitive-metabolic/inflammatory improvements induced by bariatric surgery. An understanding of such relationships may support the development of new therapeutic strategies against obesity.

## Data Availability

The datasets generated for this study are available on request to the corresponding author.

## Ethics Statement

The study was carried out in accordance with the recommendation of the ethics guideline, ethics committee of the medical faculty, University of Leipzig. The protocol was approved by the same ethics committee. All patients gave written informed consent in accordance with the Declaration of Helsinki.

## Author Contributions

MR, SvP, SH, TS, and BP designed the study. MR, SvP, SiP, SH, JH, TS, and AD performed the experiments. MR, KG, KM, and MH analyzed the data. MR and BP prepared figures, tables and wrote the article. All authors approved the final version of this manuscript.

## Conflict of Interest Statement

The authors declare that the research was conducted in the absence of any commercial or financial relationships that could be construed as a potential conflict of interest.

## Footnotes

^1^http://restfmri.net/forum/index.php?q = rest

^2^https://fsl.fmrib.ox.ac.uk/fsl/fslwiki/

^3^https://www.cbs.mpg.de/institute/software/lipsia/download
